# Change in disease activity after vitamin D supplementation in children with systemic lupus erythematosus and juvenile dermatomyositis

**DOI:** 10.1186/1546-0096-10-S1-A13

**Published:** 2012-07-13

**Authors:** Angela B Robinson, Eveline Y Wu, Egla C Rabinovich

**Affiliations:** 1 Duke University Medical Center, Durham, NC, USA; 2 Rainbow Babies and Childrens Hospital, Cleveland, OH, USA

## Purpose

Recent studies have implicated low vitamin D levels with greater disease activity in pediatric systemic lupus erythematosus (SLE) and we have reported an association between low vitamin D levels and greater disease activity in juvenile dermatomyositis. The objective of this study was to explore whether standardized vitamin D supplementation of low levels of 25-hydroxyvitamin D [25(OH)D] might be associated with improvements in disease activity.

## Methods

Subjects diagnosed with JDM or SLE prior to the age of 18 were consented and given a questionnaire regarding demographics, sunscreen use, and dietary habits to assess conventional risk factors for vitamin D deficiency. Serum 25(OH)D was determined. Disease activity was rated using a 10-cm physician’s global assessment (PGA) in all subjects and a SLE disease activity index (SLEDAI) in subjects with SLE. All subjects with serum 25(OH)D levels less than 30ng/mL were given ergocalciferol 50,000 IU weekly for 8 weeks, and serum 25(OH)D levels, PGA, and SLEDAI were reevaluated at their next routine follow up visit. T-tests and linear regression were used to examine the association between change in PGA or SLEDAI with change in serum 25(OH)D levels after supplementation.

## Results

There were 58 subjects; 21 with JDM and 37 with SLE. At baseline, 31/37 or 84% of subjects with SLE had serum 25(OH)D < 30 ng/mL, and 13/21 or 62% of subjects with JDM had serum 25(OH)D < 30 ng/mL. 23 subjects with SLE and 11 subjects with JDM had followup serum 25(OH)D levels and disease activity scores available for analysis. Among all 34 subjects, there was an overall association between decrease in PGA and improvement in serum 25(OH)D after supplementation. For every 1 point improvement in 25(OH)D, there was a mean decrease in PGA by 0.07 cm (95% CI 0.01-0.13cm) (p = 0.02). Looking independently at subjects with SLE and JDM, there was a trend towards an association between decreased PGA or SLEDAI and better response to supplementation, but these associations were not significant.

## Conclusion

Results are suggestive that there may be an improvement in disease activity associated with better response to vitamin D supplementation in children with SLE and JDM. This pilot study can be used only for hypothesis generation; it is underpowered to look at possible confounding by other medications or other factors, but suggest that a larger study to look prospectively at the effect of vitamin D supplementation on disease activity in these two diseases is warranted.

## Support

Duke Children’s Miracle Network.

**Table 1:** Mean change in disease activity compared by serum 25-hydroxyvitamin D sufficiency or insufficiency after treatment

	Follow up 25(OH)D < 30 ng/mL	Follow up 25(OH)D >= 30 ng/mL	p-value
**Overall mean change in PGA (n=34)**	0.1 cm	-1.6 cm	0.08
**Mean change in PGA among SLE (n=23)**	0.2 cm	-0.6 cm	0.47
**Mean change in PGA among JDM (n=11)**	-0.2 cm	-3.0 cm	0.15
**Mean change in SLEDAI (n=23)**	-1.6	-4.3	0.36

## Disclosure

Angela B. Robinson: None; Eveline Y. Wu: None; Egla C. Rabinovich: None.

